# A Case Study of an SMS Text Message Community Panel Survey and Its Potential for Use During the COVID-19 Pandemic

**DOI:** 10.2196/28929

**Published:** 2021-11-03

**Authors:** Lilian Chan, Nouhad El-Haddad, Becky Freeman, Blythe J O'Hara, Lisa Woodland, Ben Harris-Roxas

**Affiliations:** 1 Prevention Research Collaboration Sydney School of Public Health and Charles Perkins Centre The University of Sydney Camperdown Australia; 2 Centre for Primary Health Care and Equity Faculty of Medicine University of New South Wales Sydney Australia; 3 Population and Community Health South Eastern Sydney Local Health District Darlinghurst Australia; 4 School of Population Health Faculty of Medicine University of New South Wales Sydney Australia

**Keywords:** data collection, mobile phone, short message service, tobacco, COVID-19, survey

## Abstract

During the COVID-19 pandemic many traditional methods of data collection, such as intercept surveys or focus groups, are not feasible. This paper proposes that establishing community panels through SMS text messages may be a useful method during the pandemic, by describing a case study of how an innovative SMS text message community panel was used for the “Shisha No Thanks” project to collect data from young adults of Arabic-speaking background about their attitudes on the harms of waterpipe smoking. Participants were asked to complete an initial recruitment survey, and then subsequently sent 1 survey question per week. The study recruited 133 participants to the SMS text message community panel and the mean response rate for each question was 73.0% (97.1/133) (range 76/133 [57.1%] to 112/133 [84.2%]). The SMS text message community panel approach is not suited for all populations, nor for all types of inquiry, particularly due to limitations of the type of responses that it allows and the required access to mobile devices. However, it is a rapid method for data collection, and therefore during the COVID-19 pandemic, it can provide service providers and policymakers with timely information to inform public health responses. In addition, this method negates the need for in-person interactions and allows for longitudinal data collection. It may be useful in supplementing other community needs assessment activities, and may be particularly relevant for people who are considered to be more difficult to reach, particularly young people, culturally and linguistically diverse communities, and other groups that might otherwise be missed by traditional methods.

## Introduction

There is a high level of interest in communities’ experiences and needs during the COVID-19 pandemic [1-4], and as people’s lived experiences have been heterogeneous, more information is needed to understand what different subgroups within populations have been through. This is especially true of minority populations who are underrepresented in mainstream conversations. Traditional methods of collecting participant data, such as intercept surveys or focus groups, are not feasible during a pandemic due to physical distancing requirements.

Establishing community panels that provide data through SMS text messaging is a potential method that could be used during the COVID-19 pandemic to provide information for support services, policy planning, and research studies. SMS text message community panels allow longitudinal data collection and involve the recruitment of a sample of the community to form the community panel, and then sending a small number of survey questions via SMS text messages to the panel participants at regular short time intervals. Panel participants respond to the survey questions by sending a short response back via an SMS text message. This method allows tailoring of language to different community groups, and is particularly suitable to younger people who have been socialized to communicate using this channel. SMS text message surveys have been trialed in health research studies for data collection and have been found to be user-friendly and produce reasonable response rates [5-9].

While there are other noncontact methods of data collection that are useful during the COVID-19 pandemic, such as online surveys [10-13] and online focus groups [14], we propose that SMS text message community panels could be an additional useful tool. This report highlights an example of an SMS text message community panel, and then discusses how the approach could be used during the pandemic.

## The “Shisha No Thanks” SMS Text Message Community Panel

### Overview

“Shisha No Thanks” is a co-design project that aims to raise awareness about the harms of waterpipe (shisha) smoking among young adults (aged 18-35 years) of Arabic-speaking background in Sydney, New South Wales, Australia [15,16]. To evaluate the campaign, a community panel using SMS text message–delivered survey questions was established to identify changes in attitudes about the harms of waterpipe smoking. The survey questions were specifically designed for this study to measure awareness of project messages and attitudes toward risks of waterpipe smoking, and questions were adapted from the Cancer Institute NSW Tobacco Tracking Survey [17] and the Syrian Center for Tobacco Studies Narghile-Waterpipe Users Survey [18]. As 94% of young people (18-29-year olds) have smartphones [19], SMS text message–delivered survey questions were considered to be a useful way of engaging the target audience.

### Recruitment Process

Recruitment advertisements directed people to an online recruitment survey which was built using Qualtrics software. The recruitment survey had information about the study, and then asked for demographic information, a mobile phone number, and consent to participate in the study. People who completed the online recruitment survey were then added to the SMS text message community panel database.

To recruit and retain participants, financial reimbursements were provided. The recruitment material explained to participants that they would be compensated for their time with e-gift cards valued at AUD 50 (US $37) each, which would be sent via SMS text messages at 3 different stages of the study if they answered 75% or more of the questions. To provide context, AUD 50 (US $37) is equivalent to 6.5% of the minimum weekly wage in this country [20].

### SMS Text Message Survey Development

SMS text message community panel members were then sent 1 survey question per week. In total, the study survey consisted of 22 questions—a set of 8 questions that were asked at the beginning of the study period, 6 other questions, and then the initial set of 8 questions were asked again. This was designed for longitudinal follow-up of the cohort, to detect changes in attitudes and awareness about the harms of waterpipe smoking before and after the project.

The survey questions were also set up using Qualtrics, which allows for questions to be sent via SMS text messages. While there are many tools available for SMS text message surveys, Qualtrics was selected for this study, as it is a platform available through research institution licensing, allows for secure data hosting arrangements, and provides a user-friendly process to build SMS text message surveys (as it uses the same interface as the one used to build online surveys). It is worthwhile to note that SMS text message distribution in Qualtrics is an add-on feature, and not part of its standard license.

Each question was set up as an individual survey, and an identification number was assigned to each participant to match his/her responses throughout the study. The survey questions were designed specifically for SMS text messages, with short and concise questions that can be responded to using either multiple choice or short-text answers. Study participants were able to participate in English or Arabic (see Textboxes 1 and 2 for examples of the survey questions in English and Arabic).

**Questions from the Shisha No Thanks SMS text message survey (English language version).**

How would you rate smoking shisha compared to cigarettes considering its health effects?

1. Same
2. Less harmful
3. More harmful
4. Don’t know

What’s the main reason(s) you smoke shisha (in a few words):

Have you recently talked to someone (eg, family or friend) about the harms of smoking shisha?

A. Yes

B. No

C. Don’t know

**Questions from the Shisha No Thanks SMS text message survey (Arabic language version).**

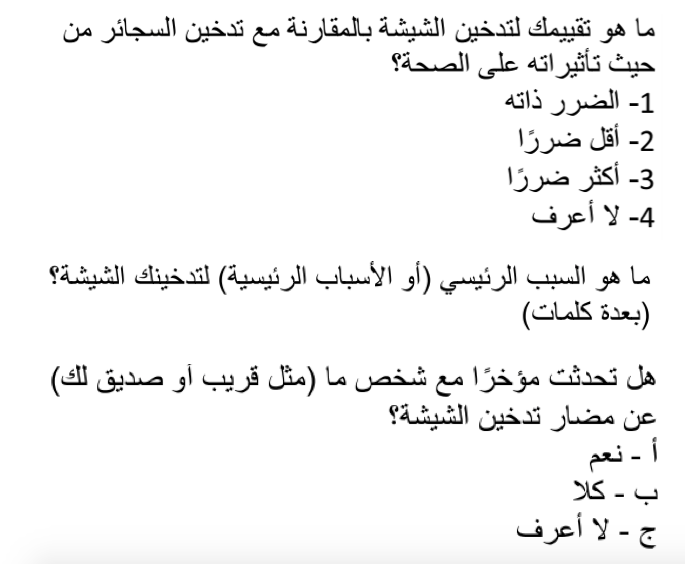

### Recruited Community Panel

The study was able to recruit 133 participants for the SMS text message community panel. This was roughly equivalent to the sample size that the project had planned for (n=100 paired responses for each response, anticipating that not all participants would respond to every question).

Community panel participants were recruited through the local community partner’s communication channels, including email newsletters and social media pages; through active local community champions who shared the recruitment survey link with their own networks via email, SMS text message, or in person; and through printed flyers with the survey link at community events, such as tertiary education open days. The recruitment survey was available on a tablet device for participants to complete at these events. Local community partners and active community champions were provided with all the recruitment materials that were used to promote the online recruitment survey.

The research team perceived the following factors to be influential in the recruitment and retainment of community panel participants to the study: nature of the study by reducing the burden of participation, participants’ age range, close engagement with the community during recruitment, participants being financially compensated for their time, and providing Arabic translations for individuals who do not speak English or prefer to participate in Arabic. Although standard SMS text messaging rates applied to the participants to answer each question, this did not hinder their response rate.

The SMS text message community panel participants’ age ranged from 18 to 35 years (mean 25.8 [SD 5.1]), with 64.7% (86/133) being female. In terms of language spoken at home, 12/133 (9.0%) spoke only Arabic, while 87/133 (65.4%) spoke English and Arabic. These demographics were consistent with the target group the research was designed to study. Only 5/133 (3.8%) participants opted to complete the survey in Arabic.

### Response Rates

The SMS text message community panel participants received questions on their phone via SMS text messages. To respond to the question, they sent their response by replying to the same number via SMS text message and typing in either a multiple-choice response or a short text. The mean response rate for individual survey questions was 73.0% (97.1/133) (range 76/133 [57.1%] to 112/133 [84.2%]). This response rate is comparable to the rates reported in other studies using SMS text message surveys [6-9]. Table 1 shows the response rates for each question that was asked before and after the project. Response rates for 6 out of 7 questions were lower for the second round.

**Table 1:** Response rates for each survey question.

Question^a^	Participants who responded (N=133)	
	First round (before project), n (%)	Second round (after project), n (%)
Q1	101 (75.9)	89 (66.9)
Q3	105 (78.9)	87 (65.4)
Q4	103 (77.4)	87 (65.4)
Q5	112 (84.2)	85 (63.9)
Q6	106 (79.7)	93 (69.9)
Q7	76 (57.1)	93 (69.9)
Q8	105 (78.9)	87 (65.4)

^a^Q2 has not been included, as it was only sent to participants who answered yes to Q1.

### Study Design Challenges

The main challenge encountered was related to the Arabic translation of participant material, including the participant information and consent form, online recruitment survey, and the SMS text message survey questions. An accredited translator translated these materials from English to Arabic. To check for accuracy and content, an Arabic-speaking researcher on the evaluation team compared the translated version with the original English version. However, during the initial recruitment phase, some Arabic-speaking participants who chose to complete the online recruitment survey in Arabic informally reported to the project officer that the participant information and consent form included complex research terminology that were difficult for the general community to understand in Arabic. To rectify this, the participant materials were re-translated using a different translation service and reviewed by 5 Arabic-speaking community members.

## Potential Uses and Benefits of SMS Text Message Community Panels During COVID-19

The SMS text message community panel is a feasible approach that overcomes many barriers to data collection during a pandemic. SMS text message community panels allow for noncontact data collection, which is an important attribute during the COVID-19 pandemic, with physical distancing and isolation being key behavioral strategies in preventing COVID-19 spread. SMS text message community panels are potentially able to include people who are more difficult to reach using other data collection methods, such as people from culturally and linguistically diverse backgrounds and differing levels of language proficiency, young people, people who live in rural and remote locations, and people with no fixed address. The method allows for timely data processing, as the data are automatically populated into digital format for analysis, which is particularly pertinent during COVID-19 as situations change quickly. This approach could be used for relatively quick data collection on needs, perceptions, and self-reported behaviors in the context of COVID-19. It is not intended to be a replacement for disease surveillance activities, but represents a potentially important additional method of collecting data.

## SMS Text Message Community Panels in Comparison to Other Data Collection Methods

In comparison to online surveys, SMS text message surveys are more specifically tailored to mobile phones. Participants respond to SMS text message survey questions in the same phone app on which they receive the questions, and the app is built into the functionality of mobile phones. By contrast, for online surveys, participants are required to click on a link in an email or social media post, which takes them to a web browser application to complete the survey. While this is a small obstacle, removing any obstacle is beneficial for improving response rates. In addition, SMS text message enables people with mobile phones (not smartphones) to participate.

Online surveys are usually developed as 1 survey with numerous questions. SMS text message community panels send only 1 or 2 questions per week, which means that participants only require a short amount of time to respond to the question(s) each week. In this way, SMS text message community panel surveys are more beneficial for measuring repeated measures at short time intervals. As an example, questions could collect data on how people are feeling during each week, and track changes in relation to situational changes (eg, small outbreaks, changes in lockdown policies, or vaccine rollout announcements).

An important benefit of using SMS text message is that it does not rely on proprietary messaging platforms, such as Facebook or WhatsApp. These proprietary platforms may have privacy or data governance implications. SMS text messages also allow a degree of anonymity as participants can be identified only as their phone number, unlike proprietary platforms, which automatically display names and personal information.

The SMS text message survey method is not appropriate to address all areas of research, particularly those that require more in-depth and detailed inquiry. Closed questions are generally limited to simple ordinal or categorical responses, and responses to open questions are limited to 160 characters before they are split up into multiple messages.

## Considerations for Use

### Recruitment and Participant Demographics

The experience of the “Shisha No Thanks” project in recruiting SMS text message community panel members demonstrated that recruitment to an already engaged community is effective. Recruitment by texting random mobile numbers with invitations to an SMS text message panel may not be effective. The researchers propose that recruitment to SMS text message community panels should be through channels where people have already established an interest or relationship (eg, signed up to community organization’s database), or through regular recruitment methods (eg, advertisements in newsletters, social media, and personal networks). In addition, this method of data collection is most suitable for recruiting participants from demographic groups who are confident with using SMS text message technology, and frequently use their mobile phone, such as the young people (18-35-year olds) who were the focus of the “Shisha No Thanks” project.

### Incentives to Participate

In the “Shisha No Thanks” project, participants reported that reimbursements acted as reminders for people to respond to the survey. Distributing reimbursements using the same platform as was used for the survey questions also facilitated this approach. During COVID-19, easily redeemable reimbursements may be equally important, given the disruptions and other challenges people face.

### Data Privacy and Security

In the “Shisha No Thanks” project, data collected were in a nonidentifiable format, and the platform used to create the SMS text message survey used firewall-protected systems and passwords to protect the data. However, SMS text message technology does not use end-to-end message encryption, and so SMS text messages do carry a risk of unauthorized access to the data. Therefore, this method of data collection may not be suitable for sensitive data. Despite the security limitations of SMS text message technology, it is worth noting that studies have found most people do not have privacy or security concerns with using SMS text messages [21,22].

### Reducing Barriers

Not everyone will have high levels of literacy, health literacy, or digital literacy. As with all surveys, careful and considered design of the survey questions can help reduce some of these barriers. Enabling the option for people to participate in the “Shisha No Thanks” study in English or Arabic presented a range of challenges including ensuring accurate translation and difficulties in ensuring non-Roman characters displayed correctly when sent via SMS text message. However, the ability to address these challenges illustrates that if inclusiveness is meaningfully considered in design, SMS text message community panels can broaden participation when compared with cross-sectional surveys, as they can be more specifically tailored to the subgroup of interest.

### Costs

An important consideration is whether the cost of SMS text message would be a potential barrier for participation. If this method is intended to be used with socioeconomically disadvantaged groups, then this issue needs to be investigated, and it may be important to use toll-free response numbers.

### Limitations

This paper presents a case study demonstrating how SMS text message community panels were used for the “Shisha No Thanks” project. Being only 1 case study, there are several limitations in our understanding about how SMS text message community panels could work for other research, particularly in the COVID-19 context. The “Shisha No Thanks” project featured substantial community participation and engagement, which may have contributed to its ability to recruit and retain participants to the SMS text message community panel. It is unclear how important this initial engagement with the community is to the success of SMS text message community panels. In addition, it is not known how important reimbursements were to the recruitment and retention success of the “Shisha No Thanks” SMS text message community panel. As reimbursement practices vary substantially between studies [23], establishing SMS text message community panels in the future with reduced or no reimbursements would demonstrate whether substantial financial reimbursements are an essential component for this method of data collection. Finally, while we did notice some attrition during the study, further research is needed to identify how long people would be willing to be engaged on such SMS text message community panels.
